# Treatment of Diffuse Alveolar Hemorrhage in Systemic Lupus Erythematosus Patient With Local Pulmonary Administration of Factor VIIa (rFVIIa): A Case Report

**DOI:** 10.1097/MD.0000000000000072

**Published:** 2014-09-19

**Authors:** Iehab B. Alabed

**Affiliations:** Internal Medicine Department, Al Hammadi Hospital, Riyadh, Saudi Arabia (IBA).

## Abstract

Diffuse alveolar hemorrhage (DAH) is a rare serious life-threatening complication in systemic lupus erythematosus (SLE) associated with a high mortality rate.

The old standard treatment options include high-dose corticosteroids, cyclophosphamide, and plasmapheresis, which are unspecific, treating the underlying disease rather than the complication itself, and not effective.

We report a case of DAH complicating SLE flare-up in a female patient treated with recombinant activated factor VII (rFVIIa) administered via the bronchoscope that showed clinical and radiological improvement.

No toxicity or adverse events were observed with rFVII treatment. rFVII may be an effective treatment option for DAH in SLE patient.

## INTRODUCTION

Diffuse alveolar hemorrhage (DAH) is a potentially catastrophic pulmonary complication with mortality rates exceeding 50% in those who require mechanical ventilator support.^1^ The hemorrhage originates in the pulmonary microvasculature, rather than from the bronchial circulation or parenchymal abnormalities.

DAH is characterized by damage to the alveolar–capillary basement membrane and alveolar inflammation, allowing red blood cells and inflammatory cytokines to enter the alveolar spaces.^2^

Most frequently, DAH is a symptom of pulmonary capillaritis as seen in autoimmune diseases or after hematopoietic stem cell transplant. Further, the damage may be the result of physicochemical factors such as blast lung injury, toxic drug effects (eg, cytotoxic drugs and crack cocaine inhalation), and radiation therapy.

The clinical syndrome is characterized by hemoptysis, falling hematocrit, hypoxemic respiratory failure, and diffuse pulmonary infiltrates.^3^

Active lupus nephritis with hypoalbuminemia is a major risk factor. Alveolar hemorrhage may occur despite ongoing treatment with corticosteroids and immunosuppressive therapy. Patients with poorly controlled disease can have recurrent episodes of DAH.^2^

Formerly, the management strategy was limited to the optimization of each of the specific diseases and underlying disorders.

Few case reports and case series that were published recently showed good results on using recombinant activated factor VII (rFVIIa) locally, which promotes local formation of thrombin when it combines with tissue factor exposed at the level of the endothelium.^4^

## CASE REPORT

This case is of a 37-year-old African woman who was diagnosed to have systemic lupus erythematosus (SLE), lupus nephritis class IV based on positive serology, and kidney biopsy treated with mycophenolate mofetil 1 g per oral twice daily that was increased to 1 g 3 times daily 1 month prior to presentation and prednisolone 25 mg once daily (0.5 mg/kg).

She presented with 5 days of shortness of breath, fever, and retrosternal pleuritic chest pain with no history of hemoptysis initially, accompanied with oxygen desaturation, which got progressively worse.

**FIGURE 1:**
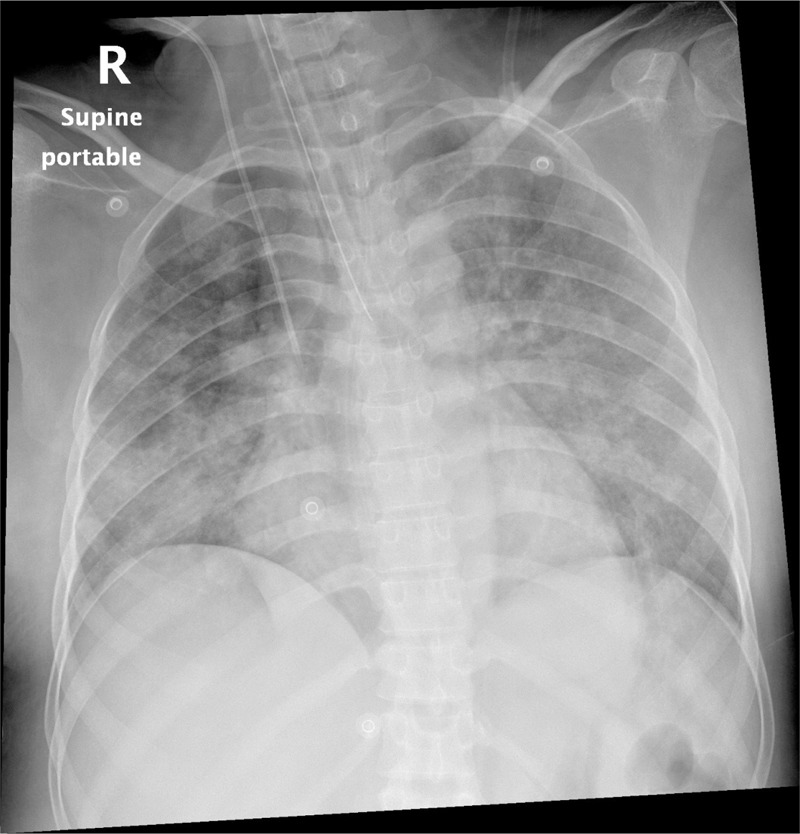
A patchy infiltrate affecting both lungs.

**FIGURE 2:**
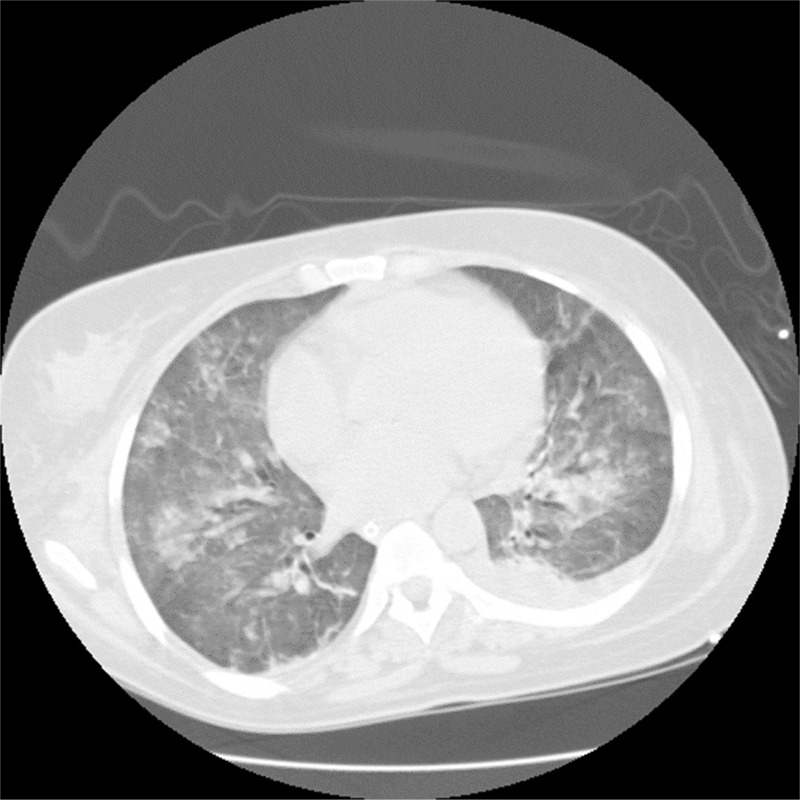
CT-chest revealed diffuse alveolar opacities. CT = computed tomography.

The patient was admitted to the intensive care unit with hypoxemic respiratory failure, requiring orotracheal intubation and mechanical ventilation. The thorax x-ray showed a patchy infiltrate affecting both upper and lower lobes bilaterally (Figure 1). Computed tomography of the thorax revealed diffuse alveolar opacities in a central and perihilar distribution indicating pulmonary bleeding (Figure 2).

Values of hemoglobin decreased from 9.8 to 8 g/dL, the platelet count was 213 × 10^9^/L, and the activated thromboplastin time was 40 seconds.

**FIGURE 3:**
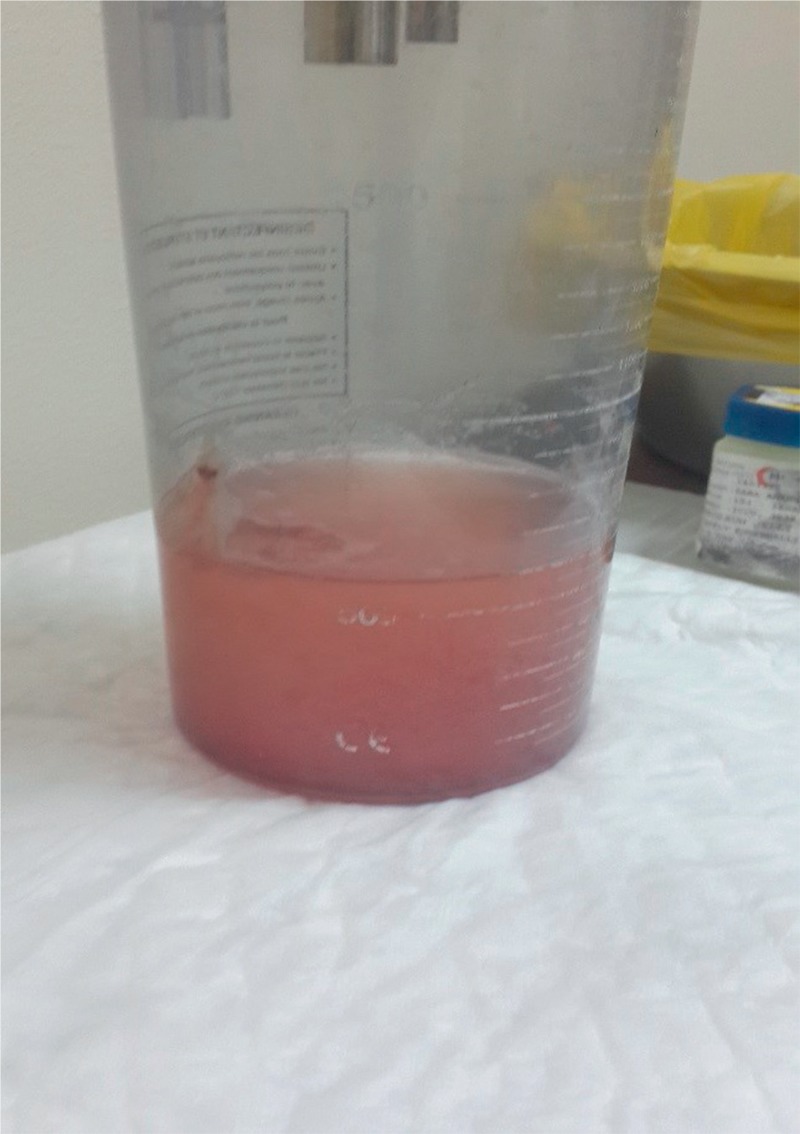
Bloody secretions from endotracheal tube suctioning.

Patient initially received systemic coverage of broad-spectrum antibiotics in conjunction with pulse treatment of methylprednisolone 1 g intravenously once daily for 3 successive days, followed by 1 dose of intravenous cyclophosphamide 1 g to be given once monthly. However, pulmonary bleeding persisted in variable degrees and tracheal suctioning was intermittently hemorrhagic; sometimes fresh blood was coming from the endotracheal tube (Figure 3).

**FIGURE 4:**
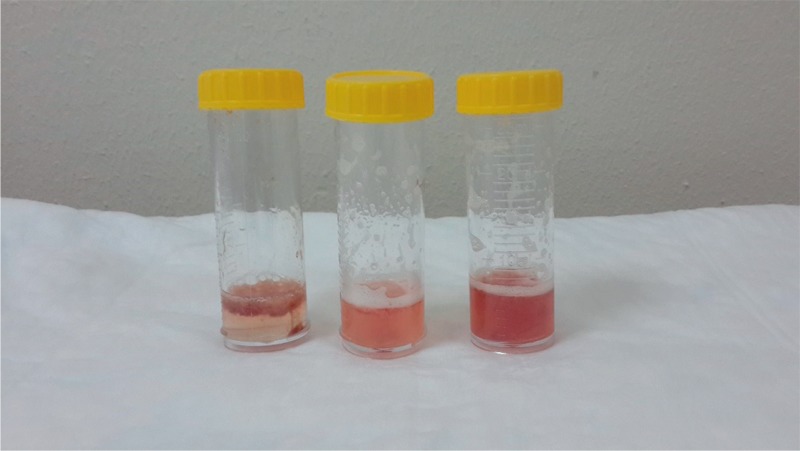
Sequential BAL samples. BAL = bronchoalveolar lavage.

**FIGURE 5:**
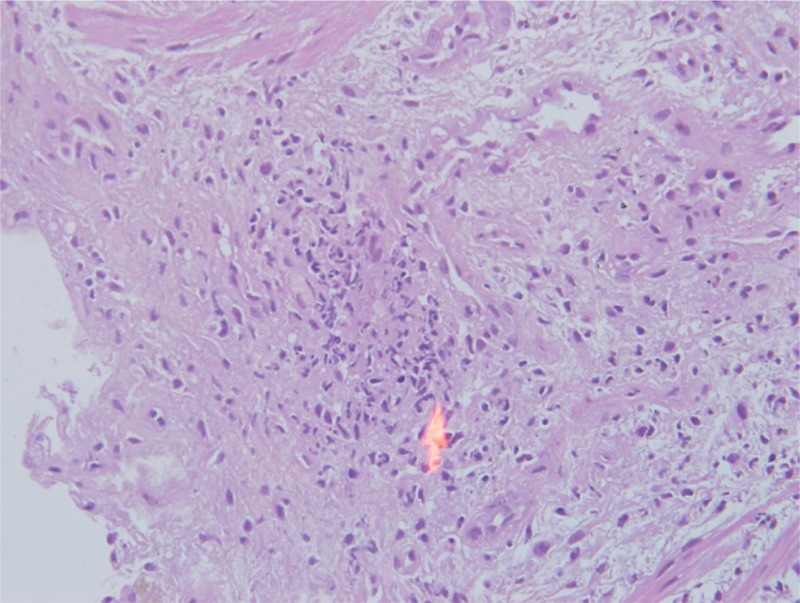
Neutrophils infiltrate blood vessels.

**FIGURE 6:**
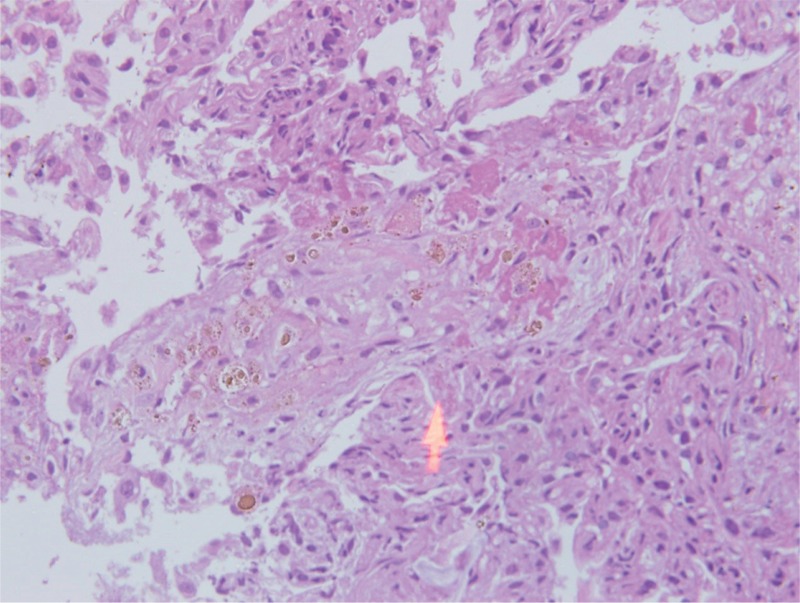
Many hemosiderin laden macrophage cells.

Fiberoptic bronchoscopy was performed and the bronchoalveolar lavage (BAL) return was increasingly bloody from both lungs (Figure 4). DAH was confirmed by BAL as a source of hemorrhage and identification of ongoing bleeding from the distal bronchial tree bilaterally. Specimens for bronchoalveolar lavage, acid fast bacilli stain, and cultures and cytology were obtained. Bronchial biopsy showed capillaritis (Figure 5). Many hemosiderin laden macrophages were with fibrin depositions consistent with DAH (Figure 6).

**FIGURE 7:**
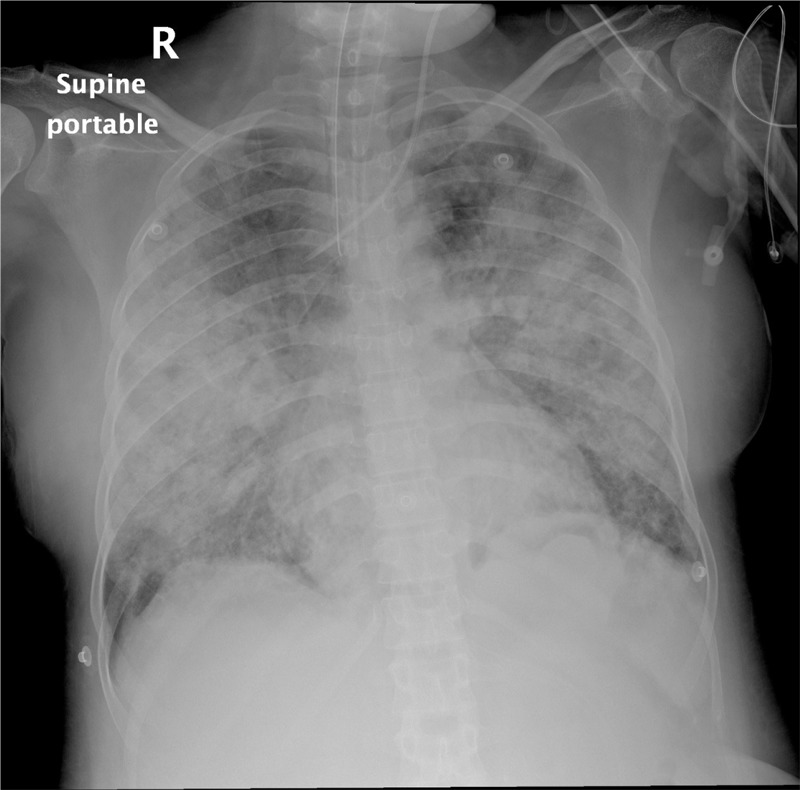
Diffuse alveolar infiltrate with butterfly pattern.

The continuation and deterioration of the patient condition clinically and radiologically (Figure 7) prompted us to decide to administer 75 μg/kg of rFVIIa in 50 mL of isotonic saline via the bronchoscopy channel, 25 mL in each main bronchus; following this, immediate cessation of bleeding was observed. Prior to this, the family had been informed and their written consent obtained.

The fraction of inspired oxygen demand could be reduced from 0.8 to 0.4 over the following 24 hours. Hemoglobin readings remained stable and did not require recurrent blood transfusion. Chest x-ray showed resolving infiltrates over several days. Weaning from ventilator was retarded due to muscular weakness.

## DISCUSSION

DAH is a clinical syndrome characterized by bleeding into alveolar spaces due to disruption of the alveolar–capillary basement membrane. Clinical features include dyspnea, cough, hemoptysis, abnormal chest x-ray with bilateral alveolar infiltrates, and hypoxia usually accompanied with fever.^5^

DAH can be caused by autoimmune disorders (eg, systemic vasculitides, Goodpasture syndrome), drug reactions (eg, propylthiouracil, amiodarone, methotrexate, nitrofurantoin, and infliximab), cardiac disorders (eg, mitral stenosis), coagulation disorders caused by diseases or anticoagulant drugs, and bone marrow or solid organ transplantation.

The treatment of DAH is empiric in as much as the condition is a life-threatening medical emergency with no specific or proven effective therapy. Treatment with high-dose steroids may be beneficial when given early,^6,7^ but overall mortality remains high; plasmapheresis has been advocated, but has not been proven to be as effective as other autoimmune causes such as Goodpasture syndrome and some vasculitis-related pulmonary bleeding.^8–10^

It is crucial to separate between treatment of alveolar bleeding and treatment of underlying disease. Local intrapulmonary administration of rFVIIa is considered as an adjuvant therapy to the underlying disease.

We report a case of a patient presented with relatively common complaints and had rapid clinical deterioration requiring mechanical ventilation. DAH was verified by bronchoscopy. There was no or insufficient hemostatic effect of standard therapy.

The rFVIIa was administered at a dose of 75 μg/kg via BAL and had a significant hemostatic effect.

The pathophysiological mechanism of action of how local intrapulmonary rFVIIa restores hemostasis has been understood more recently.

The pulmonary hemostasis can be induced more effectively from the alveolar side in DAH than the endothelial side. Intravenous rFVIIa does not reach the alveoli in contrast to the airway route where a direct access to the receptor tissue factor (TF) is obtained.

Alveolar TF is demonstrated in high concentrations in inflammatory conditions. The TF-FVII complex activates coagulation factors IX and X, which results in hemostasis.

On the contrary, the tissue factor pathway inhibitors (TFPI) that are produced by alveolar macrophages may be increased 20-fold in acute lung injury and are constitutively expressed in the airspace in inflammatory conditions, secondary to alveolar inflammation. They are strong inhibitors of the local activation of factors X to Xa by the FVIIa–TF complex.

Alveolar rFVIIa in high concentration counteracts the TFPI anticoagulation. Finally, the activated TF–FVIIa complex induces a perfect balanced hemostasis, that is, sufficient fibrin deposition without interference with the oxygen transport.^11,12^

This case emphasizes that DAH should be considered in differential diagnosis of a patient with SLE presenting with any pulmonary compliant.

Along with the standard therapies, use of rFVIIa has shown promise and should be studied more in the future.

Regarding oxygen gas exchange, it may take time to improve due to increased alveolar dead space and thickening of alveolar–capillary basement membrane because of capillaritis that may be manifested by hypoxia hypercapnia respiratory failure.

No patient died or encountered adverse effects as a consequence of the local treatment with FVIIa as reported, probably because there was no detectable FVIIa passage from the air side into the blood, as evaluated by prothrombin time.

## CONCLUSION

DAH should be considered and treated as medical emergency due to the significant morbidity and mortality associated with delayed treatment. Therefore, bronchoscopy should be performed quickly to establish the diagnosis.

This case indicates that rFVIIa when administered via local intrapulmonary route may be an effective treatment option for DAH in SLE patients and has a potentially high benefit-to-risk ratio; however, further clinical studies are needed regarding the use of this novel treatment strategy in patients with DAH.
